# Bridging the climate mitigation gap with economy‐wide material productivity

**DOI:** 10.1111/jiec.12831

**Published:** 2018-12-05

**Authors:** Kate Scott, Jannik Giesekam, John Barrett, Anne Owen

**Affiliations:** 1 Department of Geography, University of Manchester, Oxford Road, M13 9PL Manchester, Great Britain, Northern Ireland, UK; 2 Sustainability Research Institute, University of Leeds, Leeds, UK

**Keywords:** climate mitigation, demand reduction, emissions savings and climate policy, industrial ecology, material productivity

## Abstract

Projections of UK greenhouse gas emissions estimate a shortfall in existing and planned climate policies meeting UK climate targets: the UK’s mitigation gap. Material and product demand is driving industrial greenhouse gas emissions at a rate greater than carbon intensity improvements in the economy. Evidence shows that products can be produced with fewer carbon intensive inputs and demand for new products can be reduced. The economy‐wide contribution of material productivity and lifestyle changes to bridging the UK’s mitigation gap is understudied. We integrate an input‐output framework with econometric analysis and case study evidence to analyse the potential of material productivity to help the UK bridge its anticipated emissions deficits, and the additional effort required to achieve transformative change aligned with 2 and 1.5°C temperature targets. We estimate that the emissions savings from material productivity measures are comparable to those from the Government’s planned climate policy package. These additional measures could reduce the UK’s anticipated emissions deficit up to 73%. The results demonstrate that material productivity deserves greater consideration in climate policy.

**Supplementary Information:**

The online version of this article (doi:10.1111/jiec.12831) contains supplementary material, which is available to authorized users.

## INTRODUCTION

Material use contributes to climate change, one of the most pressing sustainability issues humanity faces (Krausmann, Schandl, Eisenmenger, Giljum, & Jackson, 2017; Rockstrom et al., 2009; Steffen et al., 2015). Greenhouse gases (GHGs) are emitted during the extraction of resources, the processing of raw materials, the manufacturing of final goods, the use of products and eventually when disposed of as waste (Steinberger, Krausmann, & Eisenmenger, 2010). On a global scale, material use is showing limited signs of decoupling from economic growth (Wood et al., 2018a). While it is material and product demand that drives industrial emissions (Scott, Roelich, Owen, & Barrett, 2018), climate change mitigation assessments are overwhelmingly framed around an economic and technical assessment of low carbon energy technologies and improved energy efficiency (Detlef et al., 2016; Pauliuk, Arvesen, Stadler, & Hertwich, 2017), with limited attention to material and product demand (Creutzig et al., 2016).

Climate mitigation assessment methods have been dominated by integrated assessment models (IAMs) and cost‐optimisation models (of the energy system) (Creutzig et al., 2016; Mercure et al., 2016) including computable general equilibrium models (CGEs). IAMs traditionally focus on in‐situ industries and technologies that use energy, for example power stations, modes of transport and home heating, with recent exceptions where authors have integrated supply chain analysis with more conventional models, for example see Daly, Scott, Strachan, and Barrett (2015) and Arvesen, Luderer, Pehl, Bodirsky, and Hertwich (2018), Pehl et al. (2017). The conventional models tend to ignore interconnecting supply chain energy use with patterns of everyday material and product consumption for mobility, comfort, nutrition and leisure, for example (Brand‐Correa & Steinberger, 2017; Hoolohan, Mclachlan, & Mander, 2016). By not allocating emissions to goods and services they become embodied in, this accumulation of emissions in materials and products to meet society’s needs does not enter decision making processes. For example, while only 9% of emissions produced in the EU are emitted from service and manufacturing sectors directly — and therefore would not be highlighted as a priority for mitigation in these models — they embody 22% and 26% of emissions respectively to satisfy EU consumption as they are significant procurers of GHG‐intensive materials and products along their respective supply chains (Scott et al., 2018). Taking a supply chain or embodied perspective therefore identifies further intervention opportunities in the way products are designed, sold, used and disposed of (Scott et al., 2018), which we define in this paper as material productivity measures.

Economic‐based analyses tend to reduce behaviour changes to a maximising utility function which assumes agents ‘carry out an exhaustive ranking of their preferences over all possible products in all existing markets’ (Mercure, Pollitt, Bassi, Viñuales, & Edwards, 2016), without considering other intrinsic attitudes and habits or external societal and institutional factors (Jackson, 2009). Consumers (both intermediate and final) are considered as rational actors (Levine, Chan, & Satterfield, 2015; Spence & Pidgeon, 2009; Wong‐Parodi, Krishnamurti, Davis, Schwartz, & Fischhoff, 2016). For example, firms are assumed to ‘minimise their costs by choosing an optimal combination of intermediate inputs’ (Duarte et al., 2016), which presumes companies always make fully rational choices holding all the relevant information in front of them to exhaust all options to maximise the efficiency/ reduce the impacts of their supply chains. Households are assumed to behave as a single homogenous agent, acting to maximise their utility (i.e. buying as much stuff as they can at the least cost), holding full information on all goods offered on the market to make rational choices, yet we know real world decision making deviates from the principles of rational choice (Mccollum et al., 2017; Mercure et al., 2016; Scrieciu, 2007). Such simplified assumptions have resulted in policies falling short of achieving their technical potential because of unrealistic assumptions around human behaviour (Whitmarsh et al., 2009; Wong‐Parodi et al., 2016). Therefore, current dominant models are unlikely to adequately model the mitigation potential of material productivity measures as they largely fail to understand how emissions become embodied in material and products and they have unrealistic representations of consumer behaviour.

To overcome the methodological drawbacks of climate assessments described here some scholars apply qualitative or case study evidence to simulate consumer behaviour based on quantitative consumption‐based modelling techniques such as footprinting (Barrett & Scott, 2012; Wood et al., 2018b). A GHG footprint is:

“the direct and indirect greenhouse gas emissions … required to satisfy a given consumption. This can be a product, an activity or a set of products or activities” (Minx et al., 2009).

GHG footprinting is a complementary indicator to monitor and assess progress towards meeting climate targets and can appraise the mitigation potential of demand‐side measures (Barrett et al., 2013; Scott & Barrett, 2015). Studies using footprinting or life‐cycle based techniques (e.g. material flow, life‐cycle and input‐output analysis) have demonstrated that improved material productivity is worth pursuing, if not a necessary precondition, for achieving global climate mitigation goals (Barrett & Scott, 2012; Cooper et al., 2017; Girod, Van Vuuren, & Hertwich, 2014; Liu, Bangs, & Muller, 2013; Milford, Pauliuk, Allwood, & Müller, 2013; Pauliuk and Müller, 2014). In spite of this, interventions to improve material productivity have yet to be seriously considered as an effective policy response (Scott & Barrett, 2015). Options being discussed in the literature include infrastructures being utilised more effectively through shared assets (increased asset utilisation); using products for longer (product longevity); designing products with fewer material inputs (product design); and reducing absolute levels of consumption (reducing consumption) and waste (waste reduction). These require both redesign in industry and lifestyle changes.

Input‐output (IO) models, which calculate GHG footprints at the macro scale, offer a framework to assess the economy‐wide effects of changes in technologies and consumption patterns (Wood et al., 2018b). IO models trace emissions along supply chains from production to the final demand for products by following the monetary purchases and sales of sectors (Skelton, Guan, Peters, & Crawford‐Brown, 2011; Wiedmann, 2009). This process monitors the pull and push effects of sectors on an economy (Wu & Zhang, 2005). Pull effects describe the consumption of a large amount of intermediate sector inputs and push effects describe the demands, from both intermediate sectors and final consumers, for a sector’s output. Using IO analysis each unit of emissions from production activity is uniquely attributed to a region of final demand, through complex supply chains and avoiding double counting (Skelton et al., 2011; Skelton, 2013). The analysis is able to reflect the actual emissions intensity of industries in different countries and allocate production activity in one country by intersectoral and trade monetary transactions to final demand in another country (Giljum, Bruckner, & Martinez, 2015). Whilst it would be more accurate to trace physical flows in the transformation of goods to services, data limitations have meant that IO analysis uses aggregate monetary transactions, which are collected as part of national accounting systems (Giljum et al., 2015), as a proxy of material and product flows between economic sectors and regions (Skelton, 2013).

Some researchers have developed dynamic IO models to simulate material use and emissions to 2050 by endogenously mapping economic structures as a response to price changes (Distelkamp & Meyer, 2019; Giljum, Behrens, Hinterberger, Lutz, & Meyer, 2008; Lutz & Meyer, 2009). For example, a reduction in material inputs to a selected sector changes the price and profits, which are redistributed through the economy, or a tax is applied to material extraction industries which increases their costs and reduces demand for them. In this paper we focus on non‐price simulations, which Dietz, Gardner, Gilligan, Stern, and Vandenbergh (2009) suggests introduces a behavioural realism that is lacking in technology and economic assessments.

Wood et al. (2018b) summarise the three main options for exogenously modelling consumption‐based interventions using an IO framework: (1) changing consumption patterns including a reduction in overall consumption; (2) modifying the inputs required for production in the industry (e.g., modifying the recipes of production); and (3) reducing direct emissions through, for example, pollution control or improved efficiency. They use case study evidence to identify the potential reduction of annual flows to and from EU clothing and food sectors. Lekve Bjelle, Steen‐Olsen, and Wood (2018) assess to what extent Norwegian households can lower their GHG footprint through implementing a set of behavioural actions evidenced in the literature. Emissions and economic impacts of current behaviour are compared to a better performing alternative, then scaled up to yearly savings per household. They include an analysis of the rebound effect whereby any monetary savings to households from demand reduction is re‐spent and therefore diminishes the emissions saving without this re‐spend. Cooper et al. (2017) analyse industrial energy demand reductions achieved across product supply chains through a range of circular economy opportunities applied to a 2007 baseline. They investigate different strategies which either reduce the need for high impact inputs to produce products (‘putting less in’) or reduce the need for products (‘getting more out’). All these studies analyse one point in time, and do not account for the evolving impacts of economic strategies and decarbonisation from climate policies.

In contrast, Barrett and Scott (2012) incorporate projections of key IO variables (demand, production recipes and carbon efficiencies) into their analysis of the contribution of resource efficiency measures to meeting UK 2050 climate targets. They model changes to the material demands of both production systems and consumption patterns using IO, while reducing the emissions intensity of the electricity sector. De Koning et al. (2018) extrapolate IO variables to analyse future material demands based on technical and socio‐economic considerations including economic growth, material demands and efficiency improvements. Similarly, Wilting, Faber, and Idenburg (2008) projected the production structure of the economy by using trend analysis combined with expert opinion to identify how the production inputs of sectors might change in the future. However, none of these studies have included an analysis of the full suite of climate mitigation policies alongside resource efficiency options. This has prevented an effective policy comparison.

## RESEARCH AIMS

We use an IO framework to assess the economy‐wide mitigation potential of material productivity and lifestyle measures for the UK towards meeting its climate goals. The UK sets five yearly carbon budgets to ensure it is on track to meet an 80% reduction in GHG emissions produced within its territory by 2050 (Commitee on Climate Change, 2008), and has a suite of climate policies focusing on energy used directly in the power sector, industry, buildings and transport to meet these. At the time of analysis five carbon budgets had been set from 2008 to 2032. We measure the (1) potential to help achieve the UK’s fourth and fifth carbon budgets (2022‐2032), which are anticipated to have a shortfall given existing and planned climate policies (Department for Business Energy and Industrial Strategy, 2018), and (2) how much of the UK’s carbon budget to achieve transformative change aligned with alternative 2°C and 1.5°C temperature‐related targets will be exhausted by 2032. The novelty of our approach lies in measuring the time dependency of emissions savings. We incorporate emission reductions from existing and planned climate policies as they are deployed, enabling us to identify real additional emissions savings and the contribution to meeting longer term cumulative climate goals. We compare the savings of the material productivity strategies to the suite of energy‐focused UK climate policies.

## METHOD

We develop a time series of emissions flows associated with the production and consumption of material and product demand in the UK using an IO framework. We incorporate changes in the carbon intensity of UK production sectors; disposable household incomes; government and capital spend; and export demand using UK Government economic and emissions forecasts that are constructed using macro‐econometric models combined with policy interventions. In addition to this we then model changes in the way products are designed (production recipe) and the consumption patterns of final consumers using case study evidence. We vary the ambition of material productivity strategies and the level of adoption to explore uncertainty in potential emissions savings.

### 3.1 Analysis boundaries

**Temporal** ‐ we analyse the potential for material productivity strategies to deliver UK GHG emission reductions in addition to existing and planned climate policies, concentrating on the 4th (2023‐2027) and 5th (2028‐2032) carbon budget periods, which need further policies to bridge the anticipated mitigation gap. We include emissions from 2013, the start of the second carbon budget which is yet to be concluded, projected to 2032. At the time of analysis only five carbon budgets had been set in legislation. We also calculate the remaining carbon budget the UK has to emit from 2032 to 2050 in line with international climate objectives, and how soon these could be exhausted without further policy intervention. Our analysis takes into account changes in carbon intensities according to planned timings of technological and policy implementation; however, we assume the material productivity measures are linearly implemented from 2013 to their maximum in 2032 as there is limited information on how quickly these strategies can be deployed. Hence, we have explored different rates of ambition in material reductions and adoption informed by our case studies. The case studies demonstrate the feasibility of material productivity measures within a specific (often very local) context and are not only hindered by technology availability, but also institutional and societal barriers including a lack of policy incentives and public acceptance.

**Geographic** – to measure the contribution of material productivity strategies to meeting UK carbon budgets we are only interested in emissions savings within the UK. This is not to say we don’t think the UK is responsible for reducing emissions in other countries to satisfy its consumption demands — the UK could take on greater responsibility to reflect its global economic status (Meinshausen et al., 2015; Scott & Barrett, 2015; Steininger et al., 2014). However, we want to reflect the current political reality that UK carbon budgets will be met by reducing domestic emissions. While by definition GHG footprints include emissions embodied in imports and exclude those embodied in exports destined for final consumption elsewhere, we only include emissions produced in the UK embodied in products consumed domestically, and emissions embodied in products for export. In other words, we model changes to the way UK products are made and the demand for UK products. In our model we set imported emissions to zero and therefore only emissions within the scope of domestic targets are considered, albeit emissions would be reduced along product supply chains outside the UK. These further emission reductions in supply chains outside the UK would substantially increase the calculated domestic emission reductions.

**Products/ sectors** – in terms of material productivity strategies we focus on the design and demand for materials and goods with a high embodied GHG content: textiles, food and drink, vehicles, construction, electronics and packaging. According to our analysis, these sectors contribute 14% of the industrial emissions released within the UK territory in 2013, yet embodied nearly a quarter of UK emissions (23%) (see the supporting information available on the Journal’s website, sheet A). Underlying material productivity strategies are improvements in the carbon intensity of production from existing and planned UK climate policies, which includes residential demand for heating and travel. We do not target service sectors directly, however, we do model the use of products in the provision of services (i.e. along their supply chains).

### 3.2 Emissions embodied in the final consumption of UK products

Instead of allocating emissions to the sector in which they are physically produced (‘emissions by source’), we use the UK multiregional input‐output model (MRIO) (Owen et al., 2017) to allocate UK emissions for the year 2013 to the final product they become embodied in. These final products are consumed both in the UK and abroad by households and governments or represent large capital spend.

Goods and services are classified by 106 sectors (also referred to as product groups) according to the UK Standard Industrial Classification system (Office for National Statistics, 2009) and we aggregate the global economy into a two region model of the UK and the Rest of the World (RoW) reflecting how the UK trades in goods and services. By retaining a two‐region structure we are able to capture emissions that were exported and then reimported to the UK across international supply chains. Embodied emissions are calculated using the standard Leontief demand‐pull model. GHGs emitted directly by UK sectors are reallocated to final consumers (including exports) by following products through multiple trade and transformation steps using Equation 1:

1 $$ \mathbf{q}\kern0.28em =\kern0.28em \mathbf{e}{\left(\mathbf{I}-\mathbf{A}\right)}^{-1}\hat{\mathbf{Y}} $$

Where **q** is a vector of embodied emissions by sector, **e** the GHG intensity of UK production sectors (RoW intensities are set to zero), **I** represents an identity matrix, **A** is the technical coefficients matrix and $\hat{\mathbf{Y}}$ is a diagonalised vector of the total household, government and capital final demand in the UK and RoW, including UK goods exported to RoW. The technical coefficients matrix (**A**) accounts for the proportion of intermediate inputs, both domestic and foreign, that a sector within a country requires to produce one unit of output, also known as a production recipe. The term ${\left(\mathbf{I}-\mathbf{A}\right)}^{-1}$ is known as the Leontief inverse (**L**), which calculates the extent to which output rises in each sector derived from a unit increase in final demand.

### 3.3 Projections

We project the (1) carbon intensity of UK production; (2) level of UK household demand; (3) government and capital expenditure; and (4) demand for UK exports, using macroeconometric modelling; and (5) changing production recipes and demand patterns using case study evidence, similar to Wilting et al. (2008) and (Wood et al., 2018b). See Table Table 1 for a summary of data sources.

**Table 1:** Summary of data sources used for emissions projections

	IO VARIABLE	DESCRIPTION	DATA SOURCE
ENERGY, ECONOMY AND EMISSIONS	UK carbon intensities (e)	Based on estimated emissions savings from existing and planned climate policies, alongside energy supply analysis	BEIS energy and emissions projections (Department for Business Energy and Industrial Strategy, 2018)
	Aggregate level of household expenditure (a component of y)	Central estimate from econometric trends of real disposable household income	UK Office for Budget Responsibility’s (OBR) economic and fiscal outlook used in BEIS energy and emissions projections (Department for Business Energy and Industrial Strategy, 2018)
	Government and capital spend (a component of y)	Central projections of real UK economic growth	UK Office for Budget Responsibility’s (OBR) economic and fiscal outlook used in BEIS energy and emissions projections (Department for Business Energy and Industrial Strategy, 2018)
	Foreign expenditure on UK exports (a component of y)	Central projections of real world GDP	International monetary Fund’s World Economic Outlook until 2022 thereafter following UK GDP growth projections used in BEIS energy and emissions projections (Department for Business Energy and Industrial Strategy, 2018)
MATERIAL PRODUCTIVITY	Production recipes (A)	Reduce material inputs through redesigning differently	Case study evidence (supporting information on the Web, sheet B)
	Final consumption patterns (a component of y)	Reduce demand for final products	Case study evidence (supporting information on the Web, sheet B)

#### 3.3.1 UK energy, economy and emissions projections

Since the late 1970s, the UK Government has published projections of energy demand and supply, and in the 1990s these were extended to include projected carbon dioxide (CO_2_) and other GHG emissions (Department for Business, Energy and Industrial Strategy, 2017). The Department for Business, Energy & Industrial Strategy (BEIS) is responsible for publishing these projections annually. Within their model, demand for energy is projected using a series of econometric equations that relate energy demand to its key drivers such as economic growth, international fossil fuel prices, carbon prices, population, disposable income and the number of households. Electricity producers meet demand through aiming to maximise their returns on investment. Emissions factors convert energy demand by energy source into emissions. Demand is adjusted to take account of the policy impacts where energy demand is reduced. The energy and emissions projections estimate cumulative emissions savings from an appraisal of climate policies and are used to monitor progress towards achieving carbon budgets. Policies are categorised as expired, implemented, adopted or planned.

Using BEIS energy and emissions projections we model two climate policy scenarios to determine UK emissions from 2013 to 2032 given (1) existing climate policies are implemented and (2) known and planned climate policies are implemented (the emissions scenarios are shown in the supporting information on the Web, sheet C). UK CO_2_ emissions are projected by 109 source sectors specified by IPCC (Intergovernmental Panel on Climate Change)^1^ reporting requirements, and non‐CO_2_ GHGs by nine high level sectors, whereas the IO represents 106 sectors according to Standard Industrial Classification (SIC) used mainly in economic accounting. Despite being close in number, the classification systems are quite different. We map CO_2_ and non‐CO_2_ emissions projections to SIC sectors to develop IO‐compatible production emissions data. See the supporting information on the Web, sheets D and E, for the mapping between classification systems.

To calculate the carbon intensity of UK production sectors (**e**), the UK’s sectoral emissions (**f**) are divided by economic output (**x**) of each sector (*i*) (Equation 2):

2 $$ {e}_i=\kern0.28em \frac{f_i}{x_i} $$

For future carbon intensity projections $$ {e}_i^{t+1}, $$we use official production emissions projections by sector (*i*) at five year time intervals ($$ t+1 $$). A change in output (x) is determined by a change in final demand,^2^ using Equation 3:

3 $$ {e}_i^{t+1}=\frac{f_i^{t+1}}{\sum_{j=1}^{j=n}{a}_{ij}{x}_j+\kern0.28em {y}_i^{t+1}\kern0.28em }\kern0.28em $$

BEIS climate policy projections are underpinned by central demographic, economic and price estimates (detailed in Department for Business Energy and Industrial Strategy (2018)). The econometric trends of household disposable income (${y}_j$) are used to project levels of household spending to 2032 in the IO framework. At this stage the pattern of spend is held constant. Government and capital spend (${y}_j$) follows estimated economic growth in the UK whereas demand for UK exports follows estimates of world economic growth. Increased spending (${\mathbf{y}}^{\mathbf{t}+1}$) is modelled by the Hadamard product of the vector of the original final expenditure (${\mathbf{y}}^{\mathbf{t}}$) and a vector of rate of growth for the new year (${\mathbf{g}}^{\mathbf{t}+1}$)^3^ (Equation 4):

4 $$ {\mathbf{y}}^{\mathbf{t}+1}={\mathbf{y}}^{\mathbf{t}}\kern0.28em \odot {\mathbf{g}}^{\mathbf{t}+1} $$

The Leontief equation is used to reallocate the emissions projections by source sector to the final products they become embodied in for the two climate policy scenarios, using Equation 5:

5 $$ {\mathbf{q}}_{\mathbf{climate}}^{\mathbf{t}+1}={\mathbf{e}}^{\mathbf{t}+1}\kern0.56em {\left(\mathbf{I}-\mathbf{A}\right)}^{-1}\hat{{\mathbf{y}}^{\mathbf{t}+1}} $$

Where ${\mathbf{q}}_{\mathbf{climate}}^{\mathbf{t}+1}$is the new embodied emissions by product vector calculated at five‐year intervals from 2013 up to and including 2032 from the revised improvements in carbon intensities and growth in final demand. Annual emissions between the five‐year time periods are linearly interpolated. Changes to production recipes and the pattern of final demand are described in the next sub section and determined by the material productivity case studies.

#### 3.3.2 Material productivity scenarios

We gathered evidence from 43 case studies across the six manufactured products to indicate how they could be redesigned using less carbon intensive inputs (‘putting less in‘), or the demand for new products reduced so that we get more use out of them (‘getting more out‘) (Table 2), as done in Cooper et al. (2017) (see the supporting information on the Web, sheet B for the full descriptions). In each case we identified the consumer and supplier of the material/ product according to the 106 sectors classified in the UK MRIO and the transactions flow affected in the input‐output model. The emissions associated with the transactions flows reduce in time as climate policies are implemented.

**Table 2:** Summary of material productivity strategies

SECTOR	PUTTING LESS IN (PRODUCTION)	GETTING MORE OUT (CONSUMPTION)
CLOTHING & TEXTILES	Reduce supply chain waste through efficiency improvements in fibre and yarn production, dyeing and finishing	Dispose of less and reuse more Dispose of less and recycle more Use for longer
FOOD & DRINK	Reduce avoidable food waste in food services and hospitality sectors	Reduce avoidable household food waste
PACKAGING	Reduce weight of packaging (metal, plastic, paper, glass) Waste prevention	n/a
VEHICLES	Reduce steel, aluminium and additional weight without material or alloy changes Yield improvement (metals) in car structures through cutting techniques Steel fabrication yield improvement Reuse discarded steel products	Shift from recycling to refurbishing Car clubs Use cars longer
ELECTRONICS, APPLIANCES AND MACHINERY	Reduce steel without material or alloy changes Steel fabrication yield improvement Reuse discarded steel products in industrial equipment	Sharing less frequent electrical appliances (e.g. hoovers), power tools and leisure equipment Use for longer Remanufacturing instead of throwing away
CONSTRUCTION	Design optimisation to reduce material inputs Material substitution Material reuse	n/a
Furniture*	Reduce steel without material or alloy changes	Dispose of less and reuse more Dispose of less and recycle more

^*^Emissions embodied in furniture was less than 1 MtCO_*2*_*e therefore we include savings in the overall emissions, but do not show them in the sector level analysis*.

The level of change of the transactions flow is determined by two variables: the reduction level of material/ product use (*m*) (an indication of the material ambition of a strategy) and the rate of adoption by the consumer (*c*). A low, medium and high scenario was modelled for each case study to reflect an uncertainty range in the ambition and adoption of a given strategy. The high estimate reflects a maximum technical potential in the case of redesigning products, or demand reduction levels higher than seen in existing case studies with 100% adoption in most cases. The lower level estimate reflects case studies of proven potential with relatively lower levels of adoption in the region of 33% in most cases. The mid‐estimate reflects best case estimates with 66% adoption rate. This is similar to the technical penetration and implementation variables modelled in Wood et al. (2018b), however we model a scale on which we would expect mitigation results to sit given uncertainties in ambition and deployment, and to indicate potential beyond that which is found in current case studies. A low carbon transition will require radical reductions in the way we produce and consume materials and products and we could have been even more ambitious in some of our case studies.

For each input (row *i*) to an intermediate production recipe (column *j*) vector ${a}_{ij}$ of the A matrix affected by an intervention is defined by Equation 6:

6 $$ {a}_{ij}^{t+1}={a}_{ij}^t\kern0.28em \ast \left(1-\left({m}_{ij}^s{c}_{ij}^s\right)\right) $$

where ${a}_{ij}^{t+1}$ is the new production recipe at time $\left(t+1\right)(2032)$; ${m}_{ij}^s$ is the unique level of material/ product use of a given strategy, *s*; and ${c}_{ij}^s$ is the adoption rate of policies of a particular strategy. Each element in a column of the A matrix represents the portion of the production recipe that each industry makes to the total product. We assume that when a product is made differently and requires less spend on a particular industry, this spend is effectively reallocated to value added to ensure that the row and column sums in the IO are maintained. Each strategy has a unique factor that is a combination of an industry and product interaction. *m* and c are on a scale of 0 to 1, with 0 representing no change and any number higher than this represents a reduction in the current material use and adoption rate. For example, an *m* value of 0.1 equates to 10% material reduction, for example 10% less steel inputs to manufacture cars. This follows for the adoption rate. Likewise, the same approach applies for each sector input (row i) to a final consumer (column j) for final demand (Equation 7), for example an 0.2 *m* value equates to 20% reduction in material/ product demand, for example a 20% reduction in clothes purchased by final consumers:

7 $$ {y}_{ij}^{t+1}={y}_{ij}^t\kern0.28em \ast \left(1-\left({m}_{ij}^s{c}_{ij}^s\right)\right) $$

Embodied emissions are calculated individually for each material productivity case study, in addition to climate policies, using the standard IO equation (Equation 8):

8 $$ {\mathbf{q}}_{\mathbf{materials}}^{\mathbf{t}+1}={\mathbf{e}}^{\mathbf{t}+1}\kern0.56em {\left(\mathbf{I}-{A}^{t+1}\right)}^{-1}\hat{{\mathbf{y}}^{\mathbf{t}+1}}, $$

and emissions savings, *v*, are calculated by subtracting the new embodied emissions results from the embodied emissions of the climate policy scenario (Equation 9):

9 $$ {\mathbf{v}}^{\mathbf{t}+1}=\kern0.28em {\mathbf{q}}_{\mathbf{climate}}^{\mathbf{t}+1}-\kern0.28em {\mathbf{q}}_{\mathbf{materials}}^{\mathbf{t}+1} $$

The emissions saving is calculated for 2032 $\left(t+1\right)$ and results are linearly interpolated between 2013 and 2032. This assumes the material productivity strategies are implemented incrementally, reaching maximum implementation in 2032, whereas we were able to implement climate policies at five year intervals because the official government emissions projections had the temporal detail.

The cumulative emissions savings of ‘putting less in’ and ‘getting more out’ material productivity scenarios are calculated by implementing all strategies in one calculation for differing material/ product use and implementation rates using the standard IO calculation to avoid double counting (see the supporting information on the Web, sheet F). There are additional material productivity options that we have not been able to model, due to a lack of extrapolatable case study evidence. These include strategies to extend the lifetimes of buildings and packaging. We have not provided a comprehensive list, nor an upper bound of potential reductions from material productivity, but an estimate based on available case study evidence that can be applied within our modelling framework.

We chose not to model the rebound effect, where cost savings from reduced demand are re‐spent on additional products (Arvesen, Bright, & Hertwich, 2011; Sorrell, 2010; Sorrell, 2015), as we would expect the pricing structures to change as a result of the implementation of the demand reduction strategies, adding an additional layer of uncertainty. By allocating money saved from reducing intermediate spending on inputs to value added allows the emissions intensities of the industries to remain the same and isolates the emissions effect of a change in production recipe without considering further rebounds.

### 3.4 Progress towards longer term international climate objectives

Carbon budgets will need to be set beyond 2032 in line with global climate agreements. We compare the cumulative emissions across our climate and material productivity emissions scenarios to 2032, with cumulative emissions budgets associated with three alternative temperature‐related 2050 carbon targets:

- 66% chance of 1.5°C – Global emissions converge to an average global per capita emissions point in 2050 which does not exceed the total cumulative budget to keep average global temperature rise to less than 1.5°C
- 66% chance of 2°C – Same as above but for 2°C
- UK 80% target – the existing UK 2050 climate target is equivalent to a 50% chance of exceeding 2°C average global temperature rise, but is not reconciled with a 2°C global cumulative budget

This calculation tells us how much of the 2050 carbon budgets the UK will have emitted by 2032 according to the different policy implementation rates, and by assuming that 2032 emisson levels prevail we calculate the years till the 2050 budgets will be exhuasted.

### 3.5 Scenario summary

In summary, we model five scenarios (Table 3). Two relate to the implementation of existing and planned climate policies of the UK government, and three introduce material productivity strategies across different levels of material use (varying from low to high ambition) and adoption. The material productivity scenarios are intended to present a range of emissions savings related to uncertainty in the ambition and adoption of them.

**Table 3:** Climate mitigation scenarios modelled

SCENARIO	CLIMATE POLICIES	ECONOMIC VARIABLES	MATERIAL PRODUCTIVITY
EXISTING	Existing	Central	No policies
PLANNED	Existing and planned	Central	No policies
MP_LOW	Existing and planned	Central	Low ambition and adoption
MP_MEDIUM	Existing and planned	Central	Medium ambition and adoption
MP_HIGH	Existing and planned	Central	High ambition and adoption

## RESULTS AND DISCUSSION

Most existing climate policies focus on the power sector. If the UK implements its existing and planned climate policies, emissions embodied in the power sector will reduce to 8% by 2032, yet the UK won’t have met its legislated 4^th^ and 5^th^ carbon budgets. While energy used in manufacturing is decarbonising, additional measures that reduce demand for materials and products is needed.

We present the scenario results in five sections. These in turn show:

1. The economy‐wide emissions savings over the four yet to be completed carbon budget periods (2013‐2032). This indicates whether implementing material productivity strategies, in addition to existing and planned climate policies, can meet the UK’s economy‐wide carbon budgets;
2. A comparison of the cumulative emissions savings of economy‐wide material productivity strategies across the four carbon budgets with existing and planned climate policies. This indicates whether the scale of reductions are comparable to climate policies;
3. The emissions savings from combined ‘using less’ and ‘getting more’ strategies in the 4^th^ and 5^th^ carbon budgets (2023‐2032) compared to savings from planned (i.e. not savings from existing) climate policies. This indicates whether combined material productivity measures can save more carbon than planned climate policies;
4. Emissions savings by sector in 2032. These are presented both in absolute terms and relative to each sector’s total emissions. This indicates which sectors have the highest absolute mitigation potential, and also highlights sectors that may have comparatively low absolute emissions but save a higher proportion of them through material productivity measures;
5. The percentage of carbon budgets associated with alternatively ambitious 2050 climate targets expended by 2032, and the years left till they are exhausted, assuming emissions remain at 2032 levels. This indicates the additional emissions reductions required in future depending on the ambition of longer term targets.

### 4.1 Bridging the mitigation gap

Table 4 presents the legislated UK carbon budgets, modelled emissions under implementation of climate and material productivity scenarios, and the cumulative emissions surplus or deficit. Negative values indicate a deficit. The UK’s planned climate policies are anticipated to meet the second and third carbon budgets, however, will leave a 92 and 196 Mt deficit in the 4th and 5th budgets. Depending on the ambition and adoption rate of material productivity strategies, they are estimated to reduce the 4th budget deficit between 7 and 67 Mt (8–73%) and the 5th budget deficit between 10 and 92 Mt (5–47%).

**Table 4:** Carbon budgets, cumulative emissions and emissions surplus or deficit, Mt CO_2_e

		CB2	CB3	CB4	CB5
POLICY IMPLEMENTATION	EMISSIONS DESCRIPTION	(2013–17)	(2018–22)	(2023–27)	(2028–32)
CARBON BUDGETS	Cumulative emissions	2,782	2,544	1,950	1,725
EXISTING POLICIES	Cumulative emissions	2,657	2,424	2,081	1,958
PLANNED POLICIES	Cumulative emissions	2,655	2,393	2,042	1,921
	Budget surplus/deficit	127	151	−92	−196
MATERIAL PRODUCTIVITY ‐ LOW	Cumulative emissions	2,654	2,389	2,035	1,911
	Budget surplus/deficit	128	154	−85	−186
MATERIAL PRODUCTIVITY ‐ MED	Cumulative emissions	2,650	2,375	2,013	1,880
	Budget surplus/deficit	130	163	−63	−155
MATERIAL PRODUCTIVITY ‐ HIGH	Cumulative emissions	2,644	2,353	1,975	1,829
	Budget surplus/deficit	134	175	−25	−104

### 4.2 Comparison with climate policies

**Figure 1:**
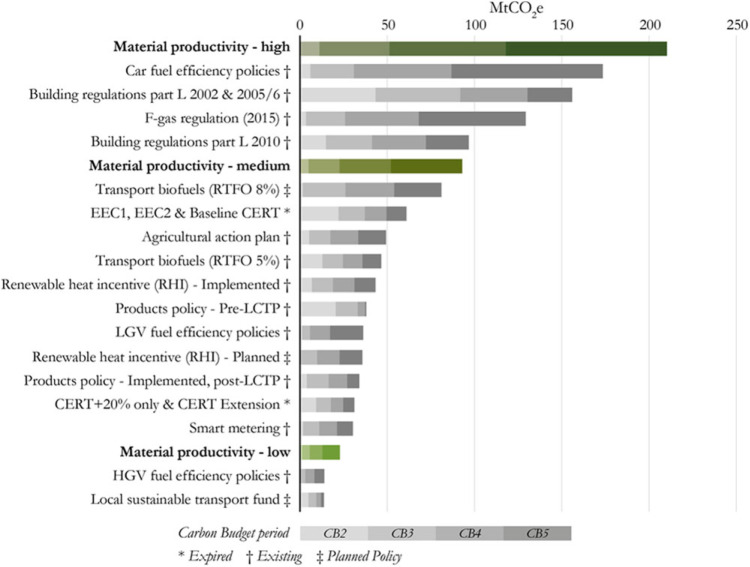
Emissions savings from material productivity scenarios compared to UK climate policy savings

Figure 1 compares the scale of potential emissions savings from material productivity strategies to those modelled by BEIS for specific climate policies over the second to fifth carbon budgets. We find that the aggregate emissions savings from material productivity strategies are comparable to those from existing climate policies and are therefore worth pursuing.

Maximum savings from the material productivity strategies we assessed across the 2^nd^ to 5^th^ carbon budgets (totalling 210 MtCO_2_e) are equivalent to those projected as a result of UK car fuel efficiency policies (173 Mt) and building regulations (156 Mt) that set minimum energy efficiency requirements to conserve fuel and power in new buildings from 2002 to 2010 (Building Regulations Part L (2002+2005/6). In our medium deployment scenario (93 Mt), savings are equivalent to the 8% target for biofuel use by diesel and petrol suppliers through the EU Renewable Transport Fuel Obligation (RTFO) (81 Mt). Taking our lowest case scenarios we anticipate savings in the region of 23 Mt, similar to those anticipated from Heavy Goods Vehicle (HGV) fuel efficiency policies and sustainable local transport plans.

### 4.3 Strategy emissions savings

**Figure 2:**
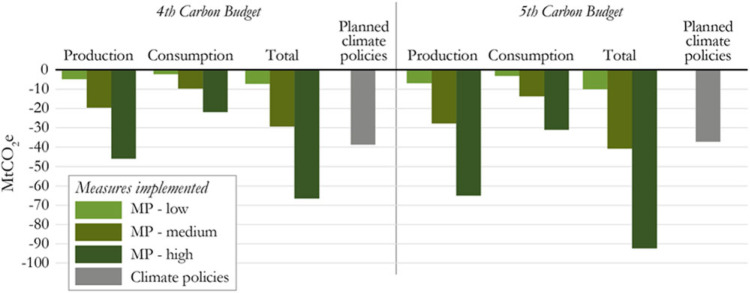
Comparison of cumulative savings from material productivity scenarios and planned climate policies for the 4^th^ and 5^th^ carbon budgets *Note: Cumulative emissions savings from production and consumption side material productivity strategies for the 4th and 5th carbon budgets are relative to the planned climate policies scenario and savings from planned climate policies are relative to the existing climate policies scenario. All savings are UK territorial emissions only*

We assessed strategies for reducing the inputs and demand for products in six sectors and compared this with emissions savings from planned climate policies (i.e. not the savings from existing climate policies) over the 4^th^ and 5^th^ carbon budget which are shown to have a deficit. This enables us to compare savings from new policies yet to be legislated. Collectively the projected savings from material productivity strategies are greater than those from planned climate policies (Figure 2). Savings from the individual strategies are given in the supporting information on the Web, sheet B, however, we focus on the cumulative savings from production (‘putting less in’) and consumption (‘getting more out’) and the 6 key sectors. A range of material productivity strategies will be needed and we are primarily exploring the economy‐wide potential of material productivity to bridge the UK’s mitigation gap.

To bridge the mitigation gap there will need to be changes to both the design of products and their consumption. This is subject to consumer preferences, business practices and policies (Barrett, Cooper, Hammond, & Pidgeon, 2018). Technical obsolescence has been designed into products for decades as a means for businesses to cut production costs and increase sales (Sherif & Rice, 1986). The low cost of products is currently a major barrier to designing and using products for longer (Cox, Griffith, Giorgi, & King, 2013). While evidence suggests that consumers exhibit preferences towards durable goods (Gnanapragasam, Cole, Singh, & Cooper, 2018), systems of production with planned (Satyro, Sacomano, Contador, & Telles, 2018) and perceived (Wieser and Tröger, 2018) obsolescence needs to be overcome.^4^ On the one hand designers can lack the expertise to create lighter or longer‐lasting products (Bakker, Wang, Huisman, & Den Hollander, 2014) whereas on the publics side shifts away from ownership of products to sharing schemes requires public acceptance of access‐based costs, yet consumers share concerns about risks and responsibilities of entering contract‐based agreements (Cherry & Pidgeon, 2018).

### 4.4 Sector emissions savings

**Figure 3:**
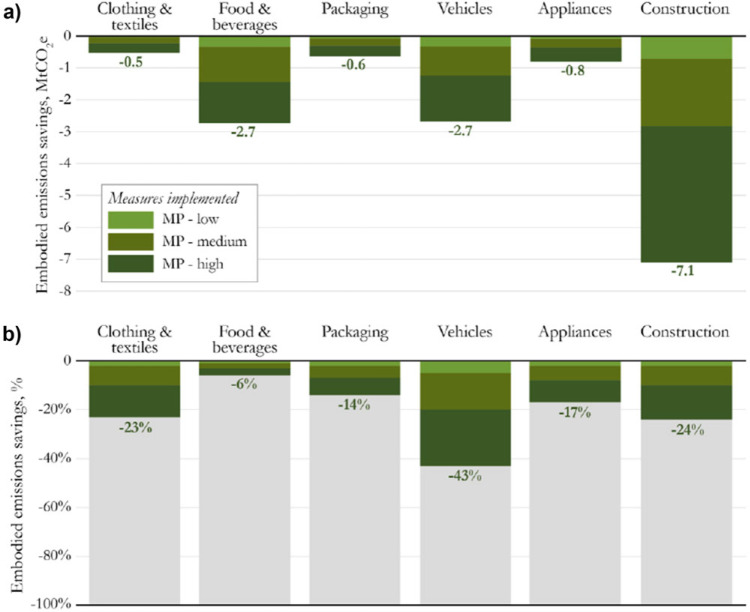
Range of territorial emissions savings by sector in 2032. a) shows absolute emissions savings and b) shows the percentage saving relative to each sector’s overall embodied emissions

Figure 3 shows the range of emissions savings embodied in our 6 key sector outputs in 2032. Emissions savings are a proportion of territorial emissions only, and not emissions embodied in imported supply chain components of these goods. In this chart we assume that decarbonisation happens in line with existing and planned climate policies. Hence, the carbon intensity of the power used to manufacture these goods is significantly reduced from today. The stacked bars represent the different deployment rates.

Planned climate policies are expected to save an additional 31Mt in 2032 compared to today’s policies. The material productivity strategies would further reduce these between 2 and 21 Mt CO_2_e (Figure 3a). This includes a 43%, 24% and 23% reduction in emissions embodied in vehicles, construction, and clothing and textiles respectively (Figure 3b).

### 4.5 Remaining carbon budget

To calculate cumulative budgets associated with the alternatively ambitious 2050 targets we assume a linear reduction in emissions from 2032 until 2050 to achieve each target. Table 5 shows the percentage of budget exhausted by 2032 for different mitigation targets and years left till exhausted assuming emissions remain at 2032 levels going forward. When compared with the UK’s legally‐binding 80% reduction target, adopting high deployment material productivity strategies, including savings from BEIS’ climate policies, will exhaust 69% of the UK’s cumulative budget by 2032, leaving 4,193 MtCO_2_e to emit from 2032 to 2050. If the UK emits the same level from 2032 onwards this budget will be used up in 10 and a half years, seven and a half years short of 2050. If the UK adopts the aspirations of the Paris Agreement to limit global temperature rise to 1.5°C, 79–80% of the UK’s 2050 budget by 2032 will have been exhausted. The budget will be used up in less than six and a half years if the UK continues to emit as it does in 2032, nearly 13 years short of 2050. Future carbon budgets will therefore need to restrict emissions greater than existing budgets.

**Table 5:** Progress towards meeting longer‐term climate targets (see section 2.4 for a description of the targets)

POLICY IMPLEMENTATION	TIMEFRAME	<66% 1.5°C CDC	<66% 2°C CDC	UK 2050 TARGET (80%)
CLIMATE PACKAGE	Budget exhausted (%)	80%	74%	70%
	Years/months till exhausted	5yr8m	8yr6m	9yr11m
MATERIAL PRODUCTIVITY‐LOW	Budget exhausted (%)	80%	73%	70%
	Years/months till exhausted	5yr10m	8yr6m	9yr11m
MATERIAL PRODUCTIVITY‐MED	Budget exhausted (%)	80%	73%	70%
	Years/months till exhausted	6 yr	8yr8m	10yr2m
MATERIAL PRODUCTIVITY‐HIGH	Budget exhausted (%)	79%	72%	69%
	Years/months till exhausted	6yr4m	9yr1m	10yr6m

Introducing material productivity measures in addition to climate policies only gives the UK extra months, not years, until these carbon budgets are exhausted. For example, if the UK adopts a 66% probability of remaining under 1.5°C the budget will be exhausted in five years eight months with the current climate package in place. Implementing maximum material productivity strategies would only buy the UK an additional eight months. This shows the enormity of the challenge and the need to think about transforming material and product use considerably more than we have modelled here.

## CONCLUSIONS

The rationale behind this modelling was to estimate the potential for material productivity strategies to bridge the UK’s territorial mitigation gap and that associated with international climate objectives. An IO framework enables us to calculate savings at the economy‐wide level, compared to more detailed bottom up studies looking at one particular product. Econometric analysis was used to project economic and emissions variables in the IO framework, allowing us to assess the interactions between material productivity measures alongside climate policies which set cumulative targets into the future. Case studies with ranges for ambition and adoption were used to simulate material productivity gains to introduce a behavioural realism, investigate uncertainty and overcome limiting behaviour assumptions related to rational choice theory.

We measured the (1) potential to help achieve the UK’s fourth and fifth carbon budgets (2022–2032), which are anticipated to have a shortfall given existing and planned climate policies, and (2) how much of the UK’s carbon budget to achieve transformative change aligned with alternative 2°C and 1.5°C temperature‐related targets will be exhausted by 2032. Emissions savings from the case studies that we modelled were comparable to those from the UK government’s existing and planned climate policy package and could reduce deficits in the 4th and 5th carbon budgets by up to 73% and 47% respectively. The stricter the climate goal in the future, the earlier we will exhaust a remaining carbon budget deemed fair for the UK. Without further changes to those we have modelled here, the UK budget for an 80% reduction target will be exhausted in as little as 10 years, which could reduce to 6 years under a 1.5°C scenario. This analysis demonstrates that material productivity deserves greater consideration in climate policy.

## Supplementary Information

**Supporting Information S1**: This supporting information provides additional data regarding 1) embodied emissions by sector in 2013; 2) material productivity strategy assumptions and individual emissions savings results; 3) emissions projections with different levels of climate policy implementation compared to carbon targets; 4) mapping IPCC CO2 sectors to MRIO SIC sectors; 5) mapping BIES non‐CO2 sectors to MRIO SIC sectors; and 6) combined emissions savings from production and consumption (excluding double counting).

## Footnotes

1 The IPCC requires only the reporting of CO_2_‐related GHGs

2 IO analysis is a demand driven model and the size of the economy adjusts to meet an increase in demand

3 **g** is a matrix representing growth across four final demand column’s spending on 106 sectors. The growth rate within each consumer’s final demand for products is the same, however the growth rate varies by final consumer

4 although this is not always the case with energy using products where improvements in energy efficiency are greater than the energy used to produce a new product
